# A duplex droplet digital PCR assay for absolute quantification and characterization of long self-amplifying RNA

**DOI:** 10.1038/s41598-023-46314-6

**Published:** 2023-11-03

**Authors:** Irafasha C. Casmil, Cynthia Huang, Anna K. Blakney

**Affiliations:** Michael Smith Laboratories, School of Biomedical Engineering, University of British Columbia, Vancouver, BC V6T 1Z4 Canada

**Keywords:** RNA nanotechnology, Expression systems, Microfluidics

## Abstract

Self-amplifying messenger ribonucleic acid (saRNA) provides extended expression of genes of interest by encoding an alphavirus-derived RNA replicase and thus is 2–3 times larger than conventional messenger RNA. However, quality assessment of long RNA transcripts is challenging using standard techniques. Here, we utilized a multiplex droplet digital polymerase chain reaction (ddPCR) assay to assess the quality of saRNA produced from an in vitro transcription reaction and the replication kinetics in human cell lines. Using the one-step reverse transcription ddPCR, we show that an in vitro transcription generates 50–60% full-length saRNA transcripts. However, we note that the two-step reverse transcription ddPCR assay results in a 20% decrease from results obtained using the one-step and confirmed using capillary gel electrophoresis. Additionally, we provided three formulas that differ in the level of stringency and assumptions made to calculate the fraction of intact saRNA. Using ddPCR, we also showed that subgenomic transcripts of saRNA were 19-to-108-fold higher than genomic transcripts at different hours post-transfection of mammalian cells in copies. Therefore, we demonstrate that multiplex ddPCR is well suited for quality assessment of long RNA and replication kinetics of saRNA based on absolute quantification.

The application of messenger ribonucleic acids (mRNA) for therapeutic or prophylactic purposes has been enabled by improving both their synthesis and delivery methods^1^. Conventional synthetic mRNA are designed to have 5’ and 3’ untranslated regions (UTRs), a poly-adenylated sequence and a gene of interest (GOI)^2–5^. Self-amplifying RNA (saRNA) is an emerging mRNA technology in which the UTRs, the replicase and the subgenomic promoter are derived from alphaviruses thus creating RNA constructs that are > 9 kilobases (kb)^6–8^. Upon entry into the cell, the positive sense saRNA is translated in the cytosol to generate the four non-structural proteins that make up the replicase complex. Utilizing conserved sequence elements present in the 5’ UTR, subgenomic promoter and 3’ UTR, the replicase complex makes copies of the genomic but substantially more of the subgenomic RNA^9^. Due to this amplification, saRNA can be administered at doses lower than the standard mRNA^10,11^. Vogel et al*.* (2018) showed that a 64-fold lower dose of saRNA than mRNA was required to protect mice challenged with the influenza virus^12^. Most recently, immunization of mice with 0.2 μg of saRNA encoding the SARS-Cov-2 spike antigen resulted in antigen-specific antibody titres as 10 μg of mRNA encoding the same antigen^13^.

Synthetic saRNA is produced similarly to mRNA, via an in vitro transcription (IVT) reaction. Although the optimization of enzyme-based IVT of long RNA is actively being pursued, production of high-quality long RNA remains a challenge^14^. Much of the focus in published literature has been on IVT reaction yield of long RNAs, but product-related impurities (PRIs), such as RNA fragments and aggregates, significantly affect downstream purification complexity and total process yield^15,16^. Commercial-scale downstream purification is required to robustly and quantifiably clear PRIs, because product efficacy and safety is related to the product quality (PQ) of the administered RNA^17,18^. Therefore, the establishment of sensitive and robust analytical assays to quantify PQ, as well as to measure PRI clearance, is required as part of the chemical and manufacturing control (CMC) regulatory submission process.

Literature on the quality assessment of relatively short mRNA molecules (~ 4,500 base pairs) is extensive^19,20^. General guidelines from the World Health Organization suggest the use of capillary gel electrophoresis (CGE) polymerase chain reaction (PCR), and analytical chromatographic methods as analytical tools to check the percentage of intact and fragmented mRNA products^21^. Notably, most of the CGE and high-performance liquid chromatography methods have been optimized and well-studied using relatively short mRNAs, but have been repurposed for quality control (QC) of saRNA despite the length differences^22,23^. Utilization of the 3’ to 5’ ratio from quantitative PCR (qPCR) provides rough and relative estimation of RNA integrity, but does not facilitate absolute quantitation^24,25^. qPCR is also more susceptible to inhibitors, and multiplexing options for multiple targets within one reaction are limited^26,27^. To circumvent the limitations of qPCR, there is a need for a robust method that enables absolute and simultaneous quantification of multiple regions of nucleic acids, such as droplet digital PCR (ddPCR).

ddPCR utilizes microfluidics to partition a PCR mixture into nanolitre-sized droplets that act as independent bioreactors for fluorescent-based quantification of nucleic acids^28,29^. Two dimensional (2D) ddPCR allows for simultaneous quantification of two targets using two fluorophores. By assuming Poisson distribution of target DNA or RNA and using a limiting dilution to single-molecule levels^28^, multiplex assays enable the quantification of both the 5’ and 3’ regions, in which the presence of both is referred to as ‘linkage’^27^. Notably, many studies have applied ddPCR as a diagnostic tool for viral^30–32^ and bacterial^33,34^ pathogens as well as clinical targets^35,36^ and gene therapy QC^37–39^. A 2D ddPCR assay was used to detect linkage between deleterious variants within the cystic fibrosis transmembrane conductor regulator gene^40^. Similarly, a 2D ddPCR was utilized to characterize the integrity of recombinant adeno-associated virus (rAAV) plasmid vectors by quantifying the ratio of double and single positive droplets. The study showed that rAAV reference standard material had ~ 60% intact genomes, which limited their infection activity^17^.

In this study, we developed a 2D ddPCR to assess the integrity of long RNA (using saRNA as a model system) through linkage analysis of distant targets. We assessed the performance of both 1- and 2-step reverse transcription-ddPCR (RT-ddPCR) in quantifying and characterizing saRNA. Gel electrophoresis, qPCR, and amplitude ddPCR multiplexing were utilized to characterize the quality of heat degraded saRNA. Moreover, 2D ddPCR was used to characterize the replication kinetics of saRNA in human embryonic kidney cells by quantifying genomic and subgenomic RNA replication.

## Results

### Assessment of PCR efficiencies and suitability of duplex ddPCR to quantify linked targets

**Figure 1:**
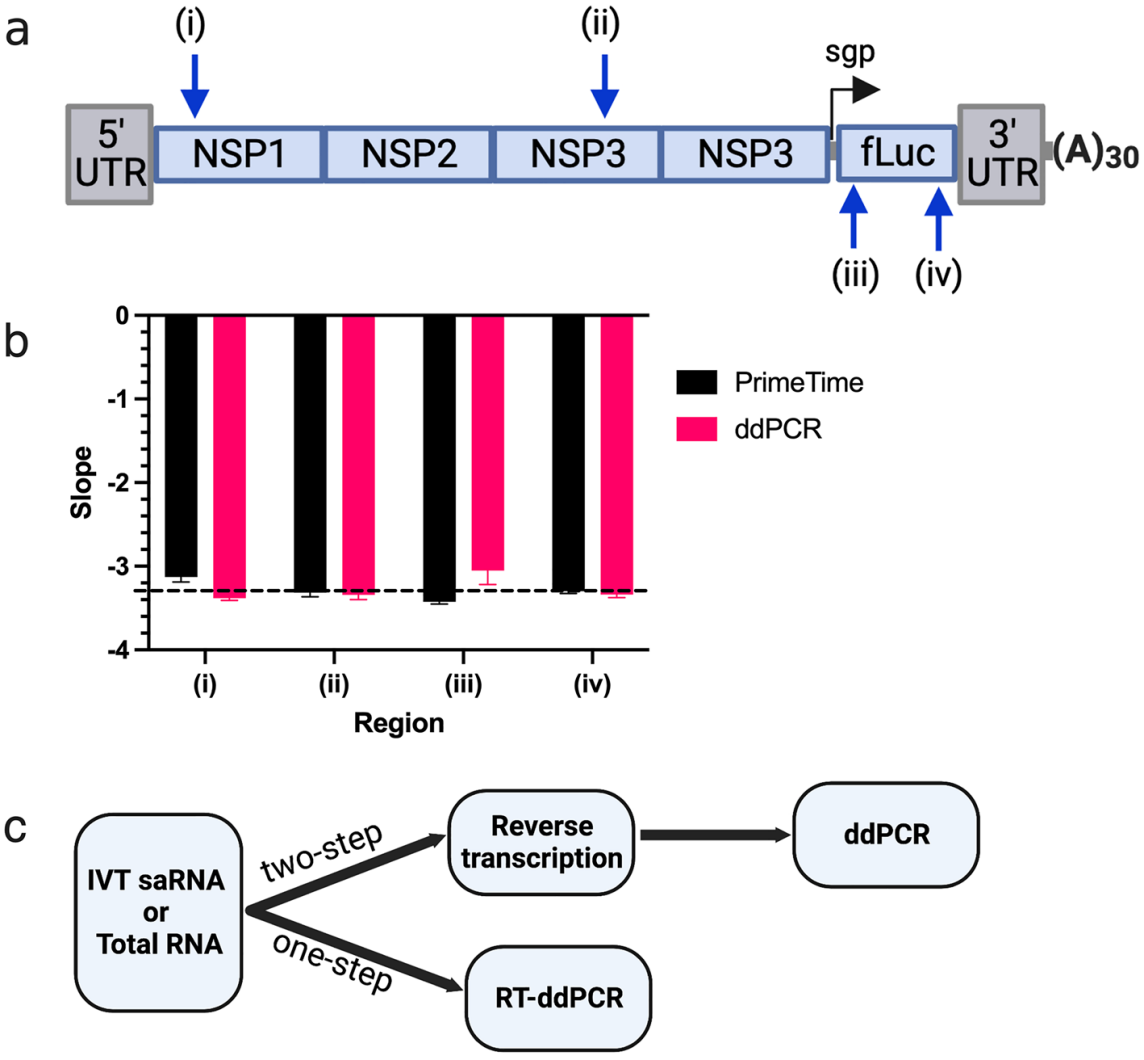
Assessment of PCR efficiency using a qPCR platform. (**a**) Map of the saRNA fLuc vector and the four regions probed: (i) VEEV 5’, (ii) VEEV nsP3, (iii) fLuc 5’ and (iv) fLuc 3’. (**b**) Bar plot indicating slopes obtained from qPCR standard experiments on all 4 regions probed using ddPCR and PrimeTime qPCR master mixes. Error bars show the standard error from 3 replicates. Dotted line shows the -3.32-slope indicating 100% efficiency. (**c**) Graphical representation of the pipeline from IVT-generated saRNA or total RNA to ddPCR assay.

We chose strategic positions within the 9,377 base pairs saRNA firefly luciferase (saRNA-fLuc) construct (Fig. 1a). Targets (i) and (iv) corresponded to amplicons on the VEEV 5’ region and 3’ fLuc, respectively, which act as proxies for full length transcripts of the saRNA construct while targets (ii) and (iii) corresponded to more central regions in the nsP3 and 5’ fLuc (Table 1).

**Table 1:** Primers and probes used for quantification of saRNA regions in ddPCR assay.

Target	Primer/Probe	Sequence	saRNA Region	Amplicon position
i	FP1^a^	CGCATGAGAGAAGCCCAGAC	VEEV 5’	9–127
	RP1^b^	TCTACCTCAAACTGCGGGAAG		
	FAM^c^	CGTTGACATCGAGGAAGACAGCCC		
ii	FP2	AGTAGGAAAAGCGCGACTGG	VEEV nsP3	4184–4304
	RP2	GGACTCATAAGCCTCTGCCAA		
	HEX^d^	CGTAGGACCAAACTTCAACAAAGTTTCGG		
iii	FP3	TCTACCCCCTGGAAGATGGAAC	fLuc 5’	7619–7743
	RP3	CTCGGCGTAGGTGATGTCCA		
	FAM	CATGAAGAGATACGCCCTGGTGCC		
iv	FP4	GCTGGAACACGGAAAGACCAT	fLuc 3’	9036–9145
	RP4	GCACCTCGTCCACAAACACC		
	HEX	ACCACCGCCAAGAAACTGAGAGGC		

^a^FP—forward primer

^b^RP—reverse primer ^c^FAM—6-carboxyfluoresceine

^d^HEX—hexachlorofluorescein.

To assess the amplification efficiency of each target region, standard curves were generated for reaction mixtures containing the qPCR PrimeTime master mix or the ddPCR supermix for probes (Supplementary Fig. S1). Standards were prepared from linearized saRNA-fLuc plasmid DNA (pDNA). Slopes obtained from the ddPCR supermix standard curve were comparable to those obtained from the qPCR master mix indicating that the PCR efficiencies for all targeted regions were within the acceptable range of -3.58 (90% efficiency) to -3.1 (110% efficiency) (Fig. 1b). After verifying the suitability of the primer/probe assays, all other experiments involving total or self-amplifying RNA underwent reverse transcription and the cDNA used in a ddPCR experiment or the RNA was used directly in a 1-step RT-ddPCR (Fig. 1c).

**Figure 2:**
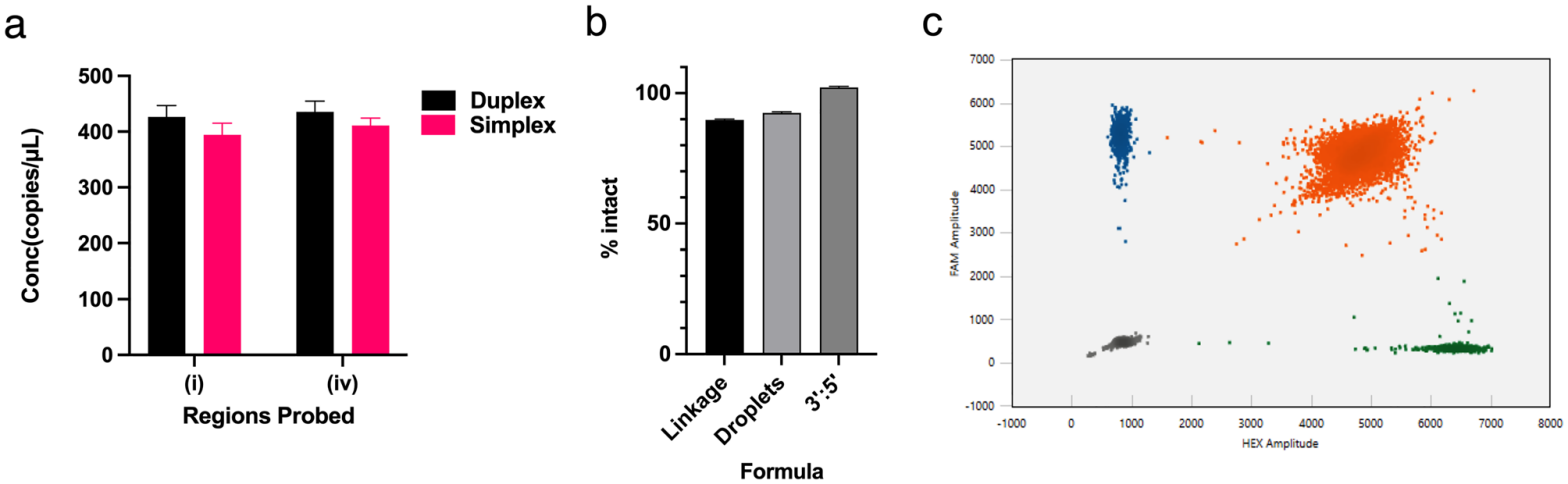
Duplex assay for quantifying full-length transcripts of saRNA fLuc pDNA. (**a**) Concentration in copies/μL of targets (i) and (ii) on pDNA as determined via duplex or simplex assays. Error bars show 95% total Poisson error (**b**) Comparison of multiple formulas used to calculate % of intact pDNA—linkage analysis, fraction of double positive droplets and ratio of 3’ to 5’ targets. Error bars show standard deviation of n = 3 replicates (**c**) A 2D amplitude plot for the duplex assay. X-axis and Y-axis show the amplitude of droplets detected in the FAM and HEX channels, respectively.

Next, linearized pDNA was used to assess the suitability of the duplex assay to quantify full length transcripts. There was no significant difference between the copy numbers obtained from duplex assay probing for the full-length sequence (targets (i) and (iv)) when compared to the simplex assay probing for the regions separately (Fig. 2a). In the duplex assay, the droplet reader detected the presence of the FAM, and HEX dyes as shown on the y- and x- axes respectively (Fig. 2c). The clusters were classified as follows: negative droplets (bottom left), FAM single-positive droplets (top left), HEX single-positive droplets (bottom right), and FAM + HEX + double-positive droplets (top right). Multiple formulas were employed to establish the most accurate way to calculate the percentage of full-length transcripts.

First, the linkage method as described in Regan *et al.* (2015) and derived in the supplementary information considers the probability that a droplet might contain multiple fragments including linked targets that might be detected^40^. The percentage of intact transcripts as shown in Eq. 1 with lambda (λ) indicating copies per droplet:

1 $${\text{\% intact}} = \frac{{\lambda_{i + iv} }}{{0.5\left( {\lambda_{i} + \lambda_{iv} } \right)}} \times 100{\text{\% }}$$

Next, the droplet formula determined the fraction of double positive droplets containing targets i and iv as shown in Eq. 2 with *N* representing the number of droplets:

2 $${\text{\% intact}} = \frac{{N_{i} N_{iv} }}{{N_{i} + N_{iv} + N_{i} N_{iv} }} \times 100{\text{\% }}$$

Finally, the 3’:5’ formula determined the percentage of the 3’ concentration over the 5’ as depicted in Eq. 3 where C denotes copies/μL:

3 $${\text{\% intact}} = \frac{{C_{{3{^{\prime}}}} }}{{C_{{5{^{\prime}}}} }} \times 100{\text{\% }}$$

It was expected that if the saRNA was full-length, all formulas would show 100%. The percentage of full length pDNA transcripts were 89.72 ± 0.34, 92.47 ± 0.35 and 102.2 ± 0.45 by the linkage, droplet and 3’:5’ formulas, respectively (Fig. 2b). These results confirmed that duplex ddPCR assays can be utilized to determine the fraction of intact nucleic acid transcripts in a sample.

### The one-step RT-ddPCR provides information on the integrity of saRNA

**Figure 3:**
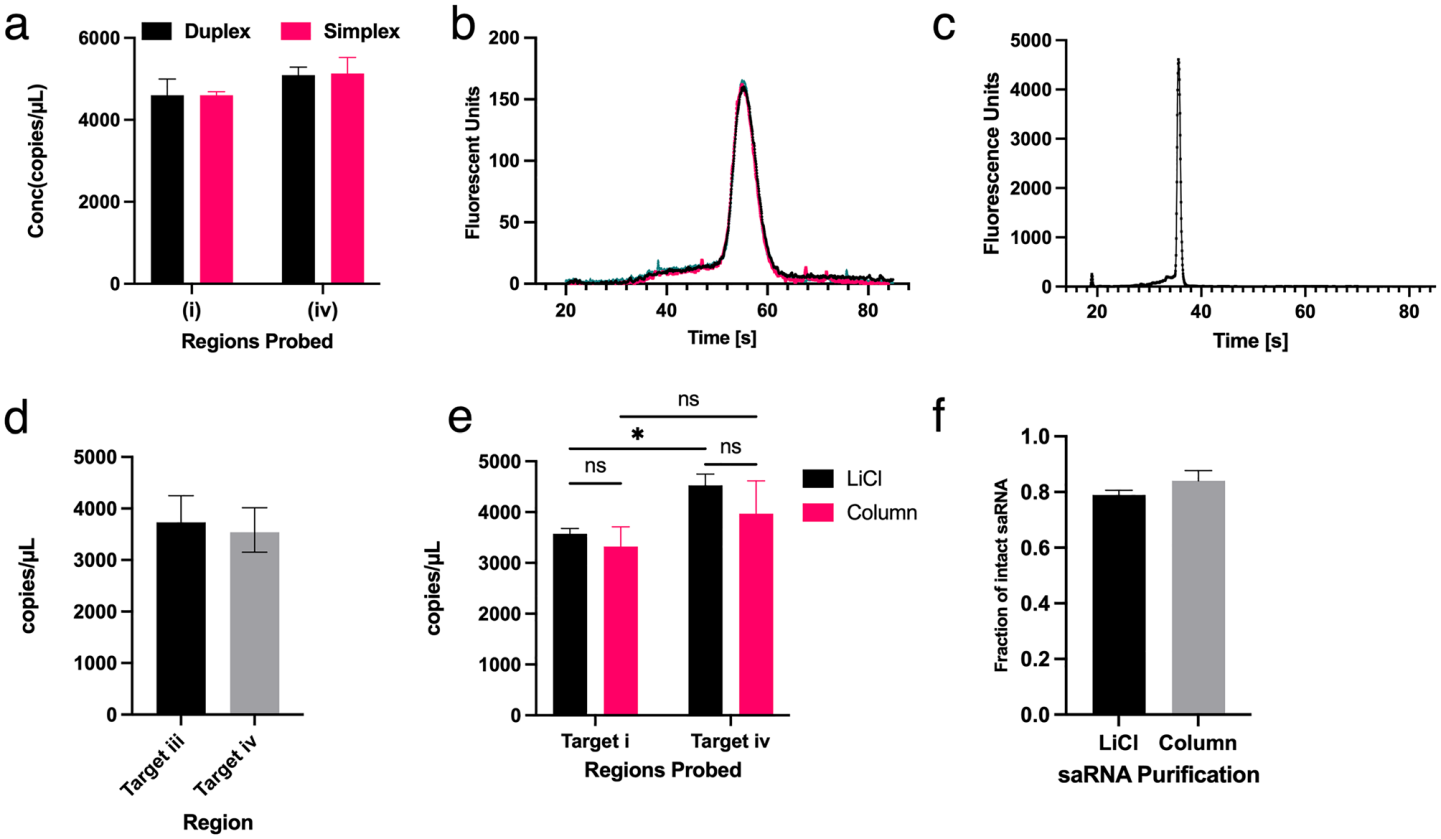
Application of the 1-step RT-ddPCR on saRNA. (**a**) Comparing the performance of the duplex and simplex assays shows similar quantities of targets (i) and (iv) are obtained. Error bars show 95% total Poisson error. (**b**) Electrogram of saRNA samples run on the Bioanalyzer. (**c**) Electrogram of mRNA samples run on the Bioanalyzer. (**d**) Concentration of mRNA regions corresponding to fLuc 5’ and 3’. (**e**) Assessment of the effect of LiCl precipitation and column purification of saRNA on RT-ddPCR assay. (**f**) Fraction of intact saRNA after two different purification methods as determined using 3’:5’ formula.

Having established the reliability of the ddPCR assay to assess the integrity of long nucleic acids using relatively stable pDNA, we sought to quantify the quality of IVT saRNA-fLuc. 1 pg of saRNA was used in a simplex and duplex ddPCR reaction probing for targets (i) or (iv) and both, respectively. 4,601 and 4,604 copies/μL of target (i) were detected in the simplex and duplex 1-step RT-ddPCR assays, respectively. Similarly, 5134 and 5094 copies/μL of target (iv) were detected in the simplex and duplex assay (Fig. 3a). Similar performances of the duplex and simplex assays indicated that multiplexing for multiple targets does not affect the performance of the assay. The linkage, droplet and 3’:5’ formulas determined that the percentage of full-length saRNA transcripts were 56.39 ± 5.53, 98.92 ± 0.15 and 110.56 ± 4.47, respectively. Although the linkage method tends to underestimate the level of intact pDNA controls, the method showed similar results when saRNA was assessed using the Bioanalyzer (Fig. 3b). Based on the trapezoid rule of determining area under the curve (Fig. S2a, c and Supplementary method), the Bioanalyzer results indicated the presence of 63% intact saRNA. The droplet and 3’:5’ methods indicated the presence of 99% and 110% of intact saRNA, respectively thus considered as overestimations of intact saRNA.

To expand the application of the 1-step RT-ddPCR, we sought to characterize mRNA-fLuc. Since mRNA-fLuc is 1,766 bp, it was unambiguously sized using the Bioanalyzer (Fig. 3c). The electrogram indicated ~ 86% intact mRNA-fLuc. The fLuc 5’ and 3’ regions were probed, and it was determined that 3,731 and 3,541 copies/μL were detected, respectively. The droplet and 3’:5’ method indicated that > 94% of the mRNA-fLuc were intact while the linkage method showed 82% intactness (Fig. 3d). Therefore, it was established that the ddPCR assay was not limited to longer RNA but was also capable to sizing relatively shorter transcripts.

Having verified that the 1-step RT-ddPCR assay can quantify both saRNA and mRNA, we sought to establish the robustness of the duplex assay. Robustness of a quantitative assay is defined as the capacity to provide consistent results when small changes are made to the experimental procedure^41^. Slight changes in the experimental design and setup were that were tested included saRNA purification and 10% reduction in components of the reaction mixture. saRNA was purified using LiCl precipitation and a silica spin column. RT-ddPCR indicated that no statistical significance was observed between the two purification methods (Fig. 3e). Upon assessing the percentage of intact saRNA obtained, column purification provided 84% of intact saRNA as compared to the 79% obtained after LiCl precipitation (Fig. 3f). Additionally, the RT-ddPCR reaction mixture was setup with a 10% reduction of the final concentration of the reverse transcriptase and the supermix. Despite the perturbations to the assay, similar levels saRNA qualities were obtained (Supplementary Fig. S3a). Therefore, we showed that saRNA QC using RT-ddPCR remained unchanged or robust in the face of small and deliberate changes to the methods.

### Choice of reverse transcriptase and priming strategy is integral in a two-step RT-ddPCR assay

**Figure 4:**
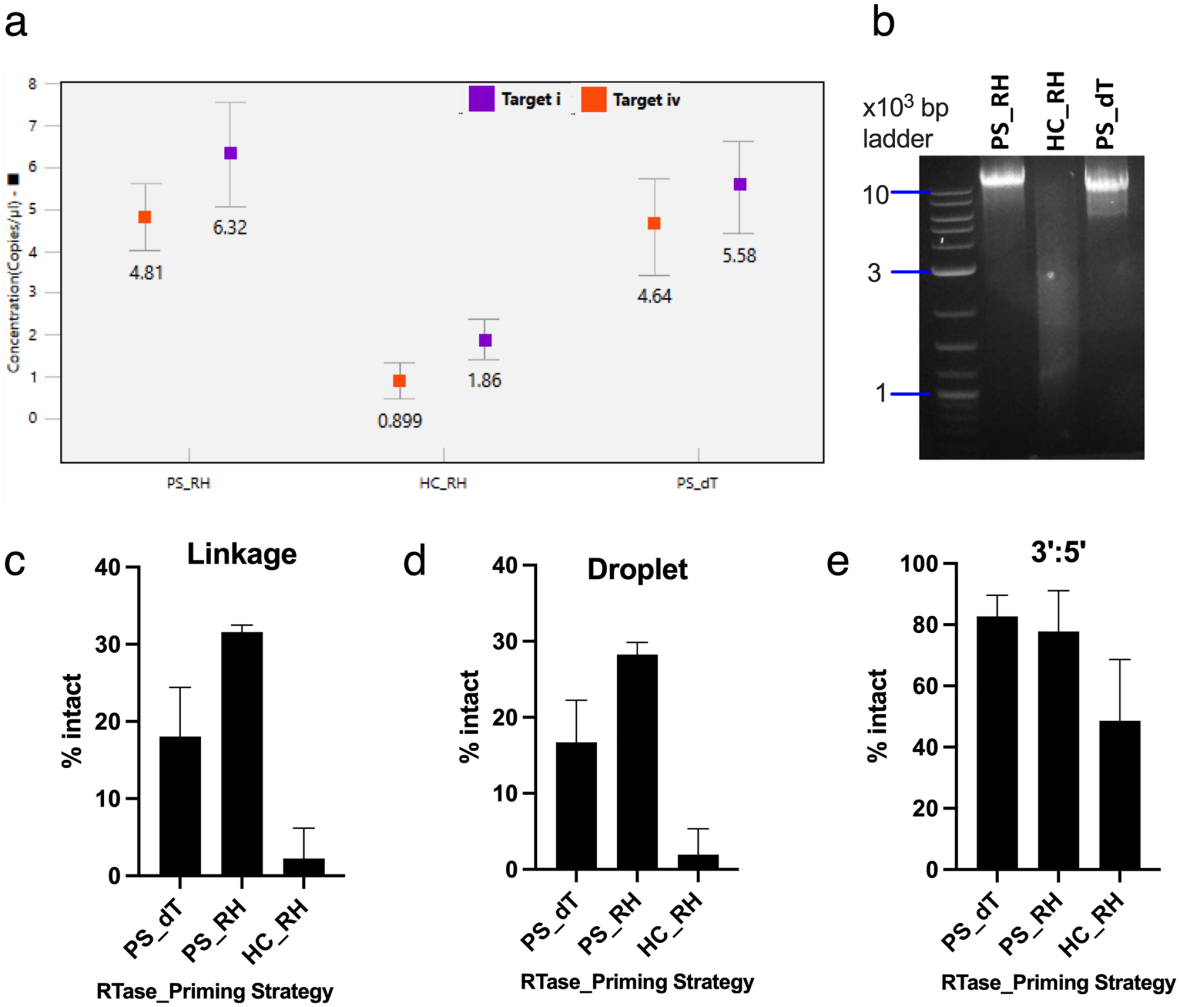
Effect of reverse transcription strategy on quantification of intact saRNA. Reverse transcription strategy abbreviated as kit_primer: PS—ProtoScript kit from NEB, HC—High-Capacity kit from Applied Biosystems, RH—random hexamers, and dT—oligo dT primers. (**a**) Equal amount of saRNA reverse transcribed using different strategies. PS_dT considered as a pseudo positive control for full length transcripts when target i is detected. Error bars indicated total Poisson error of all droplets. (**b**) Agarose gel of cDNA generated using the various kits. Original gel is presented in Supplementary Fig. S7. (c**–e**) Percentage of intact cDNA obtained from different RT reaction as determined by the linkage, droplets and 3’:5’ formulas. Bars show mean ± standard deviation of n = 3 replicates.

For increased flexibility, we compared the one- and two-step RT-ddPCR. 1 μg of IVT-generated saRNA was reverse transcribed using random hexamers (RH) or oligo dT (dT) primers and the reverse transcriptase enzymes present in the ProtoScript (PS) or High-Capacity (HC) kits. Due to the long nature of saRNA, we sought to determine which reverse transcription strategy would transcribe the full length of the saRNA. Henceforth, the reverse transcription ProtoScript or High-Capacity kit and random hexamers or oligo dT primers as PS_RH or PS_dT or HC_RH. We hypothesized that the detection of target (i) on cDNA generated using oligo dT primers would be an indication of cDNA with the same size as the fully intact saRNA. The cDNA reaction was diluted to 10^–3^ pg/μL RNA equivalent. 2.2 μL of the diluted cDNA was used in a duplex ddPCR assay probing for targets i and iv (Fig. 4a). The HC kit underperformed significantly for both targets when compared to the PS + dT. Use of random hexamers with the PS kit, generated a higher number of targets than the PS + dT reactions. These results showed that only 1.96% of positive droplets were double positive when the HC_RH kit was used as compared to the 28.26% and 16.72% obtained when using PS_RH and PS_dT kits, respectively. To verify the reverse transcription capabilities of each strategy, 5 μL of the cDNA rection mixture was run on an agarose gel to provide a qualitative analysis of the size of the cDNA (Fig. 4b). The HC_RH lane had a smear as compared to the PS_RH and PS_dT lanes that had a significant amount of cDNA above 9 kb. A similar trend was observed when the linkage and 3’:5’ formulas were applied to the results to determine the percentage of intact cDNA (Fig. 4c–e). The linkage and droplet formulas show that there is about 30% full length cDNA while the 3’:5’ formula indicates that 62–82% of cDNA is full-length. The discrepancy in the formulas can be traced back to the limitations of the reverse transcription reaction. The percentage of intact generated through the PS-RH strategy cDNA as determined by the 3’:5’ formula was within the range obtained using the 1-step RT-ddPCR. Therefore, it was concluded that the 3’:5’ ratio was appropriate to quantify the fraction of intact saRNA when reverse transcription primed using random hexamers and done in a separate step to the ddPCR. Additionally, a serial dilution of cDNA generated using the PS_RH strategy was utilized to determine the linearity of the ddPCR assay and the reproducibility of the 3’:5’ formula at different dilutions. (Supplementary Fig. S4).

### Utilization of the duplex ddPCR assay to assess the quality of heat-degraded saRNA

**Figure 5:**
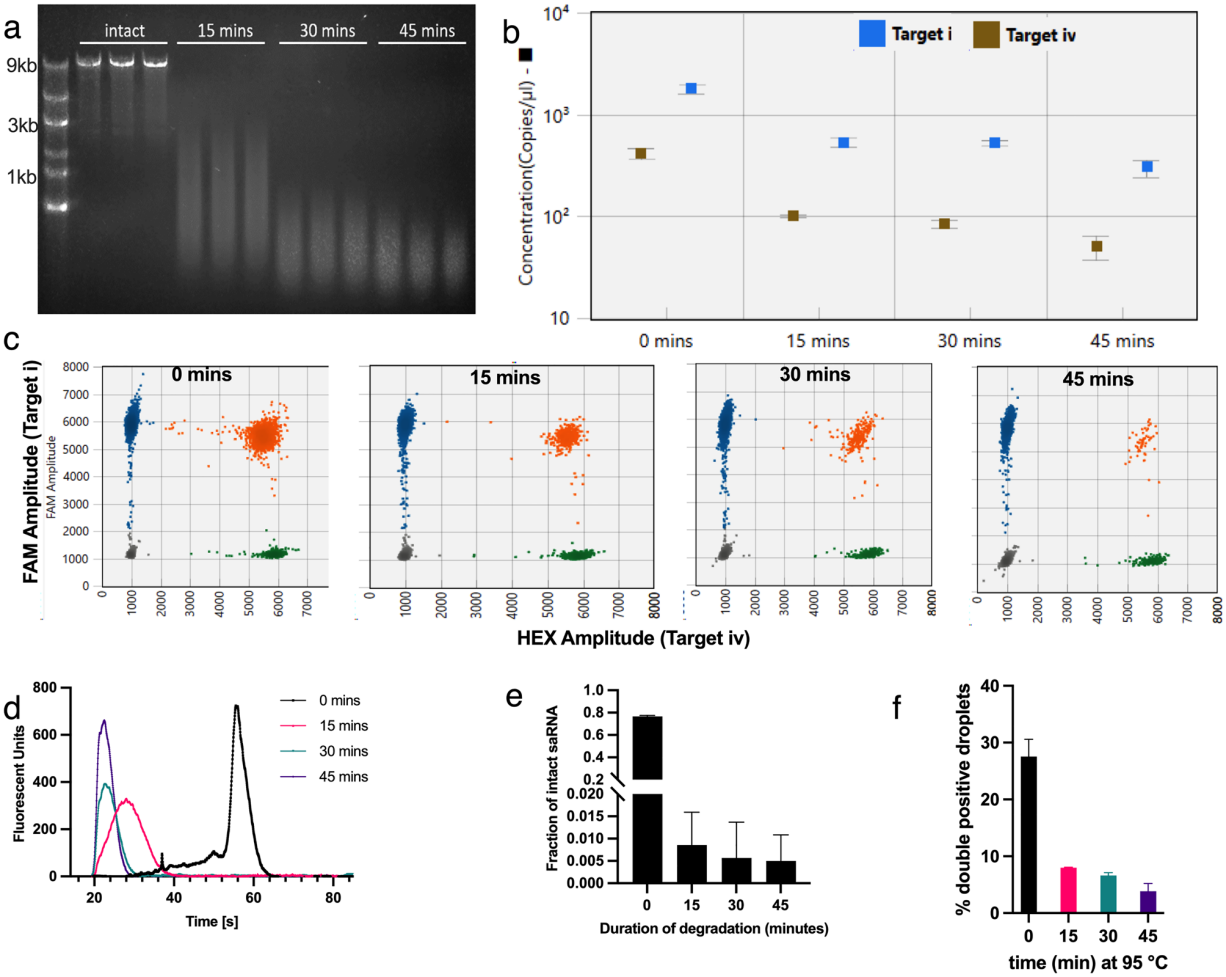
Quality assessment of saRNA degraded at 95 °C at different timepoints. (**a**) Agarose gel saRNA triplicate samples degraded for 0 (intact), 15, 30 and 45 min at 95 °C. Original gel is presented in Supplementary Fig. S8 (**b**) copies/µL detected from cDNA of heat-degraded saRNA. (**c**) 2D amplitude plot for the duplex assay on heat degraded samples. (**d**, **e**) Electrogram and fraction of heat-degraded saRNA present based on results obtained from the Bioanalyzer. Bars show mean ± SD of n = 3 replicates.

We then assessed the capability of the ProtoScript Kit paired with random hexamers to differentiate between saRNA samples that were known to be intact or degraded. saRNA was degraded at 95 °C for 0, 15, 30 and 45 min. The samples were run on an agarose (Fig. 5a) and capillary electrophoresis (Fig. 5d,e) systems. The intact saRNA (0 min of degradation) had a smear between the 3-9 kb (Fig. 5a) or the presence of the left tail on the electrogram (Fig. 5d). The smear in the IVT saRNA yield was expected as an inherent limitation of IVT of longer RNA species. Quantification of the intact saRNA based on the area of the electrogram showed the presence of 76% of intact saRNA. As the level of saRNA degradation increased, the smear on the agarose gel was augmented and the intensity of the full-length band decreased, while the respective electrograms as determined by Bioanalyzer shifted to the left and the amount of intact RNA as determined by ddPCR was negligible. cDNA from the heat-degraded saRNA was utilized in a duplex assay probing for target (i) and (iv). The concentration of both targets reduces with extended time at 95 °C (Fig. 5b). Despite, having higher cDNA copies at 0 min, target (i) had a higher degradation rate than target (iv) as evidenced by slopes of -31.73 ± 6.779 and -7.854 ± 1.707 copies/μL per minute, respectively. However, it should be noted that the linear fit for targets (i) and (iv) had R^2^ values of 0.7088 and 0.7018, respectively. Qualitatively, the 2D plot (Fig. 5c) mirrors what was observed in the electrophoresis and ddPCR experiments. As the level of degradation increases, the amount of double positive droplets reduced despite the slight plateauing of copy numbers at the 30- and 45-min degradation mark. Further assessment of saRNA degradation was performed in a triplex assay detecting targets (i), (ii) and (iv) (Supplementary Fig. S5). Triple positive droplets indicating the presence of full-length cDNA reduced as the saRNA was heated for longer periods. These results show that 2D ddPCR was a robust method for quantifying intact or degraded saRNA since the co-occurrence of targets (i) and (iv) in a duplex assay or targets (i), (ii) and (iv) in a triplex assay change accordingly.

### Characterization of replication properties of saRNA in human cell lines using ddPCR

**Figure 6:**
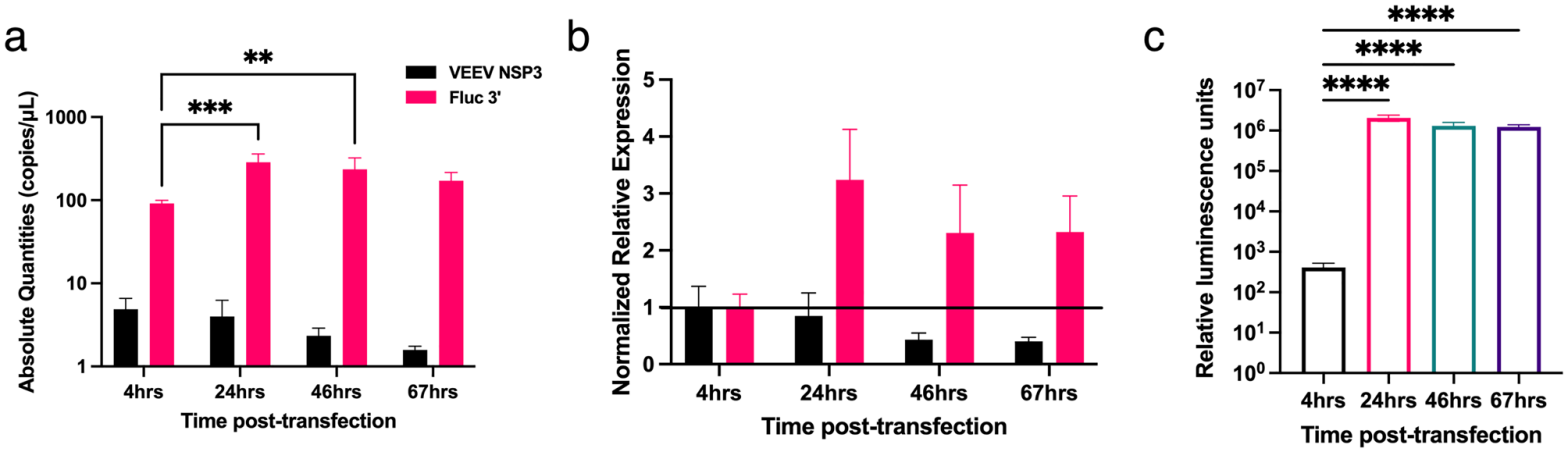
Quantification of saRNA transcripts and translation levels in 293 T cells. (**a**) absolute quantities of target ii and iii at different timepoints post-transfection. **p = 0.0021, ***p = 0.0002 by two-way ANOVA followed by Dunnett’s multiple comparison test. (**b**) Normalized relative expression of nsP3 and fLuc. Horizontal line at y = 1 indicate the threshold used as a control. (**c**) Luciferase assay at parallel time points when total RNA was collected. ****p < 0.0001 by one-way ANOVA followed by Dunnett’s multiple comparison test. Bars indicate mean ± SD of n = 3 samples.

In addition to the QC of IVT-generated saRNA, we aimed to quantify the integrity and relative replication of the genomic and subgenomic RNA present total RNA obtained from cells 4, 22, 46 and 67 h after lipofectamine transfection. At the 4-h timepoint, all media including any saRNA-lipofectamine polyplexes were washed away with PBS, enabling us to assume that no more saRNA was introduced to the cells after this time. At all-time points, the amount of the subgenomic RNA region (target (iii)) was higher than the non-structural protein region (target (ii)) indicating that the subgenome containing the fLuc gene was being replicated (Fig. 6a). Copies of target (ii) decreased throughout the experiment while target (iii) increased at 24 h and decreased thereafter. 19-, 71-, 100- and 108-fold differences were obtained between the subgenomic and genomic regions. Surprisingly, substantial replication of the sub-genome had occurred at 4 h post-transfection as indicated by the 19-fold difference the concentrations of target (ii) and (iii). All samples when compared to the 4-h time point showed that the non-structural region was downregulated while the fLuc subgenome was upregulated (Fig. 6b) after normalization (reference gene quantification in Supplementary Fig. S6). The expression of protein, as determined through a firefly luciferase assay, reflected a similar trend to the replication of the subgenomic region of the saRNA (Fig. 6c). We observed a significant increase is observed between 4 and 24 h. Afterwards, the luminescence detected decreases gradually. These results confirmed the replicative properties of saRNA through absolute quantification of the genomic and subgenomic copies present at different time points.

## Discussion

Effectiveness and potency of mRNA therapeutics is dependent on the intactness of the molecule. Here, we assessed the integrity of long saRNA produced from IVT reaction using a duplex, 1-step RT-ddPCR. Similarly, we utilized different reverse transcription setups to generate cDNA whose integrity was assessed using the 2-step RT-ddPCR. Quality assessment results obtained from RT-ddPCR were compared to other methods such as capillary gel electrophoresis and qPCR. In addition to quality assessment of saRNA, duplex RT-ddPCR was utilized to characterize the replication kinetic of saRNA in human cells.

Taking advantage of the random Poisson distribution of targets into droplets and expected 1–2 target copies/droplet at low concentrations, the frequency of double positives can be used as a proxy for intact transcripts. This approach is utilized to characterize the integrity of recombinant adeno-associated viral vectors^17^. However, it does not consider the random occurrence of double positive droplets. Alternatively, the linkage analysis method estimates the presence of intact transcripts by determining the excess number of double positive droplets as comparted to double positive droplets that occur by chance^40^. The 3’:5’ ratio is an overestimation as it considers the total concentration of each target regardless of being in single and/or double positive droplets. The 3’:5’ relative levels based on qPCR data has previously been used to indicate the level of degradation in total RNA^23,24^.

To characterize physical linkage of saRNA regions as shown in Fig. 1a, strategic target regions were chosen. Of note, we did not choose regions within the 5’ and 3’ UTR due to the conserved secondary structure elements that are present in saRNA. These regions were also avoided due to the inhibitory effects of secondary structures on the efficiency of reverse transcription which is an essential step in characterizing and quantifying RNA^42,43^. The saRNA construct used a 5’ UTR of 51 nucleotides, a 3’ UTR of 107 nucleotides and a 30-nucleotide poly A tail. However, this might not be a challenge in the case of saRNA or mRNA derivatives with longer UTRs unlike our saRNA constructs. Linkage analysis of pDNA as a positive control indicated a 10% underestimation (Fig. 2b) than the expected 100% intact pDNA. The underestimation is consistent with Shehata et al. (2017) who noted that after prolonged digestion or heat treatment of DNA templates resulted in the decrease of the ddPCR output^44^. Prolonged digestion (2 h at 37 °C) and heat treatment (20 min at 65 °C) of the pDNA template occurred during the linearization process prior to setting up the IVT reactions.

Linkage analysis, double-positive droplet characterization and the 3’:5’ analysis of the results generated by 1-step RT-ddPCR revealed that an IVT reaction generated 60–85% full length saRNA (Fig. 3a). Similar to the Bioanalyzer results (Fig. 3b), the linkage method indicates equivalent amounts of intact saRNA. Therefore, the linkage method is the recommended formula when utilizing the 1-step RT-ddPCR. The presence of shorter than the ~ 10 kb saRNA is attributed to incomplete transcription by the T7 RNA polymerase, degradation of the pDNA template or degradation during purification of the saRNA. The proportion of intact saRNA has significant implications on the amplification capabilities of any saRNA therapy. Our results consistently show fewer 3’ target copies which suggests that ~ 30% of transfected saRNA lacks the 3’ conserved sequence elements required for the synthesis of the minus strand from the positive, genomic strand^45^. In addition to being replication deficient, partially intact saRNA have hairpin motifs with double-stranded RNA sequences that induce the interferon (IFN) response pathway that led to its rapid degradation. In the case of saRNA-based therapeutics, replication and translation is to be maximized while minimizing IFN-related degradation^46,47^. Therefore, more robust technical guidelines and QC approaches are required as clinical applications of RNA technologies expand^48^.

We characterized the effect of an additional reverse transcription step on the quality assessment of IVT-generated saRNA. Multiple studies have noted that the reverse transcription step introduces variability into any quantitative reactions^49–51^. Comparing two recombinant Moloney murine leukaemia virus with (ProtoScript II reverse transcriptase, PS kit) or without (MultiScribe reverse transcriptase, HC kit) RNase H activity showed a sixfold difference in the percentage of intact saRNA. Both reverse transcription strategies had truncated products, but the MultiScribe reverse transcriptase combined with random hexamer priming was more severe (Fig. 4c–e). Consistent with reports that indicate that reduced RNase H activity and thermophilic capabilities of a RT enzyme improve production of long cDNA^52,53^, the PS kit generated cDNA as long as the saRNA (Fig. 4b). Similarly, the 2-step RT (PS_RH)-ddPCR showed a 20% difference in linkage when compared to the 1-step RT-ddPCR. The difference between the two most likely arose from the use of random hexamers and saRNA specific primers for the reverse transcription step in the 2- and 1-step RT-ddPCR, respectively. However, the 3’:5’ formula was within the range of intact saRNA as determined via the 1-step RT-ddPCR and Bioanalyzer. The outperformance of gene specific primers during reverse transcription has been reported in prior RT-qPCR studies^53,54^. Based on the results, comparing the 5’ targets detected when oligo dT and random hexamers can be used as a proxy for the fraction of full-length transcripts owing to the different saRNA species that each strategy primes for RT. Therefore, we conclude that use of random hexamers and a reverse transcriptase with reduced RNase H activity in conjunction with the 3’:5’ formula for analysis was sufficient for saRNA quality assessment. In the case of a mixed population of RNA species such as total RNA, oligo dT primers or construct specific primers would be more applicable.

Expanding the application of the duplex ddPCR, we characterized the replication of saRNA fLuc in mammalian cells (Fig. 6). The in vitro replication trajectory of saRNA in HEK293T cells showed a gradual drop of the non-structural target while the fLuc sub-genome increased, plateaued, and decreased by the 67th hour after transfection. Early timepoints of the replication have a higher amount of the non-structural region which is consistent with studies that have shown that significant anti-viral responses occur at early stages of transfection^55^. The use of absolute quantification of saRNA in conjunction to protein quantification such as the luciferase assay (Fig. 6c) provided a clear link between RNA and protein level. Expanding the application of ddPCR to quantify the intra-cellular replication of saRNA has significant application for the engineering and optimization of saRNA therapies.

In this study, we show that the multiplexing capabilities of ddPCR can be used to determine the integrity of saRNA and other long RNA transcripts generated through IVT. Absolute quantification was also applied to the replication in mammalian cell cultures and was correlated to the amount of expressed protein. The ddPCR assay can be applied to assess delivery efficiency of nanoparticle delivery vehicles by quantifying the absolute amount of RNA detected at different points of a saRNA therapy. Similarly, this assay can be applied at all stages of the development pipeline and manufacturing process for saRNA or mRNA therapies, from assessing the quality of pDNA template used for IVT, to the quality of saRNA following: IVT, capping and poly-adenylation, downstream purification unit operations, formulation, and storage stability.

## Methods

### In vitro transcription

saRNA was designed to contain a 5’ untranslated region (UTR), non-structural protein-coding regions, a subgenomic promoter (SGP), a 3’ UTR from the Venezuelan Equine Encephalitis virus (NCBI taxonomy ID: 11,038) and 30 nucleotides poly-Adenosine 3’ tail^56^. Between the SGP and 3’ UTR, a firefly luciferase (fLuc) gene (GenBank: AB762768) was inserted. mRNA-fLuc contained the 5’ and 3’ UTRs from Tobacco mosaic virus. The plasmid DNA (pDNA; 11,427 base pairs) was transformed into 5α *Escherichia coli* (New England BioLabs, USA), cultured in 100 mL of Luria Broth with 100 μg/mL carbenicillin (Sigma-Aldrich, USA) and extracted using a Plasmid Plus MaxiPrep kit (Qiagen, USA). The concentration and purity of the pDNA was characterized through absorbance measurement on a NanoDrop One (ThermoFisher, USA). The pDNA was linearized with SapI (New England BioLabs, USA) for 2 h at 37 °C and inactivated for 20 min at 65 °C. 1 μg of linearized pDNA was used in an in IVT reaction to generate capped saRNA and mRNA using the mMessage machine kit (Thermo Fisher Scientific, USA) and subsequently purified using the lithium chloride precipitation method or the MEGAclear Transcription clean-up kit (Invitrogen, #AM1908) as described by the manufacturer. Capped saRNA transcripts were resuspended in UltraPure H2O, their concentration and purity measured using the NanoDrop (Thermo Fisher Scientific, USA), aliquoted into 20 μL samples and stored at  − 80 °C until further use. The saRNA and mRNA yield per 20 μL IVT reaction was 378 ng/μL and 162 ng/μL, respectively. All RNA samples had ~ 2.19 for the A_260/280_ and 2.3 for the A_260/230_ ratios.

### Primers and probes design

The nucleotide database of the National Centre for Biotechnology Information (NCBI; https://www.ncbi.nlm.nih.gov/tools/primer-blast/) was used to design primer pairs for 4 regions spanning the saRNA construct. A PCR product length of 90–120 base pairs was chosen while activating the function to exclude any primer pairs that may anneal to the human transcriptome. Corresponding probes for each primer pair were designed using the IDT PrimeQuest tool (https://www.idtdna.com/PrimerQuest/Home/Index). The primers and probes (Table 1) were manufactured at Thermo Fisher Scientific and Integrated DNA Technologies, respectively.

For GAPDH primers and FAM-labelled probe, forward: GTCAGCCGCATCTTCTTT; reverse:CGCCCAATACGACCAAAT; probe: CCGTTGACTCCGACCTTCACCTTC.

### Complementary DNA (cDNA) synthesis

For the two-step RT-ddPCR or RT-qPCR, first strand cDNA synthesis was performed in a 20 μL reaction mixture as follows: 1 μg of saRNA was reverse transcribed using the High Capacity (Thermo Fisher Scientific) and ProtoScript (New England Biolabs, CA) kits for 2 h at 37 °C and 42 °C, respectively. Random hexamers (RH) and oligo-dT primers were used for priming the RT reaction at a final concentration of 6 and 5 mM, respectively. In the case of RH, RNA was mixed with the primers and denatured at 25 °C for 10 min before adding all other components of the RT reaction. For in vitro replication experiments, 100 ng of total RNA, primed with oligo dT primers was reverse transcribed using the ProtoScript kit as described above.

### Quantitative PCR

Reactions were performed according to the manufacturer’s protocol. Briefly, a reaction mixture of 1X PrimeTime Gene Expression master mix with ROX reference dye or the ddPCR Supermix (no dUTP) (Bio-Rad, USA), 900 nM of forward and reverse primers, 250 nM of corresponding probes and cDNA from the RT reactions. Thermal cycling was performed using the 7500 Fast Real-Time PCR (Thermo Fisher Scientific, USA) system set at the maximum ramp rate. The PCR cycling conditions using the PT master mix were as follows: polymerase activation at 95 °C for 3 min followed by 40 cycles of denaturation at 95 °C for 15 s and extension at 60 °C for 1 min. The PCR cycling conditions using the ddPCR supermix were as follows: polymerase activation at 95 °C for 10 min followed by 40 cycles of denaturation at 95 °C for 30 s, extension at 60 °C for 1 min and enzyme deactivation at 98 °C for 10 min.

### Droplet digital PCR

The one-step RT-ddPCR was performed according to manufacturer’s instructions. Briefly, a 22 μL reaction mixture of the 1X supermix, 400 units of the reverse transcriptase, 15 mM of DTT, 900 nM of forward and reverse primers, 250 nM of corresponding probes and 1 pg of RNA was made. Thermal cycling was performed on the C100 Touch Thermal Cycler (Bio-Rad, USA) at a ramp rate of 2 °C/second. The ddPCR cycling conditions were the same as described above except an initial reverse transcription step at 42 °C for 60 min. Subsequently, 20 µL of the reaction mixture was emulsified into droplets according to manufacturer’s instructions for droplet generation using the QX100 droplet generator and the QX200 droplet reader (Bio-Rad, USA). Data was acquired using the QX manager software (Bio-Rad) and exported to GraphPad Prism, version 9.4.1 (GraphPad Software) for further analysis.

For the two-step RT-ddPCR, cDNA was generated as described above. Subsequently, a 22 μL PCR reaction mixture was prepared as follows: 1 × ddPCR Supermix for probe (no dUTP), 900 nM of forward and reverse primers, 250 nM of corresponding probes and 5 μL of cDNA or linearized pDNA. Droplet generation and reading was performed as described above.

Droplet analysis was performed in the QX manager software. Thresholds for positive and negative droplets were manually or automatically set. The results from 9,000 or more droplets were used to calculate concentrations. The concentration of independent replicates was reported as copies/μL ± total Poisson error (alpha = 0.5).

### Gel electrophoresis

DNA samples and 1 kilobases plus DNA marker were mixed with 6 × loading dye (New England BioLabs, USA), loaded onto a 1% agarose gel in Tris–acetate-EDTA buffer and run at 110 V for 50 min. RNA samples were run and analysed using the FlashGel RNA cassette system (Lonza Bioscience, USA).

Additionally, saRNA samples were prepared according to the Agilent RNA 6000 Nano Kit guide and run on the Agilent Bioanalyzer 2100 using the mRNA Nano assay class protocol. The data was analysed by the 2100 Expert software (B.02.10.S1764). Fraction of intact saRNA based on the electrogram was represented by area under the curve (Supplementary information). The Lonza RNA marker (Lonza Bioscience, USA, #50,577) was used.

### Cell culture, transfection, and total RNA extraction

Lenti-X 293 T (Takara Bio, USA) cells were cultured in complete media made up of Dulbecco’s Modified Eagle Medium supplemented with 10% fetal bovine serum, 1% Glutamax, and 1% penicillin–streptomycin (Thermo Fisher Scientific, USA). 3.5 × 10^5^ cells per well were seeded in 24-well plates. 24 h after seeding, 500 ng of saRNA were transfected using lipofectamine 3000 according to manufacturer’s instructions. 4 h post-transfection, media containing saRNA lipoplexes was removed and cells washed with PBS before addition of complete growth media. At different timepoints, media was removed, cells were washed and detached, and total RNA was extracted.

Total RNA was extracted using the Total RNA miniprep kit (New England Biolabs, USA) according to the manufacturer’s guidance (including the on-column DNase I digest). RNA concentrations and purity was determined using the NanoDrop. Reverse transcription and ddPCR was performed as described above.

### Data analysis and graphing

GraphPad Prism (version 9.4.1) was used to draw graphs and perform any statistical analysis as outlined within the corresponding results.

### Supplementary Information

Supplementary Information.

## Data Availability

The data that support the findings of this study are available from the corresponding author (A.K.B), upon reasonable request.
