# Supplementary material for: A duplex droplet digital PCR assay for absolute quantification and characterization of long self-amplifying RNA
Source: Sci Rep. 2023 Nov 3;13:19050. doi: 10.1038/s41598-023-46314-6 (PMC10624827; doi:10.1038/s41598-023-46314-6)
Supplement: Supplementary file 1 — Supplementary Information. [file 41598_2023_46314_MOESM1_ESM.pdf]

A duplex droplet digital PCR assay for absolute quantification and characterization of long self-amplifying RNA

Irafasha C. Casmil[1], Cynthia Huang[1] and Anna K. Blakney[1,*]

[1] Michael Smith Laboratories, School of Biomedical Engineering, University of British Columbia, Vancouver, British Columbia V6T 1Z4, Canada

* To whom correspondence should be addressed. Email: anna.blakney@msl.ubc.ca

SUPPLEMENTARY INFORMATION

Derivation of the linkage formula

The linkage formula was derived as follows with i and iv indicating regions probed, N representing number of droplets, E representing empty droplets, λ indicating copies per droplet:

Equation 1a: occurrence of double positive droplets by chance:

$$\text{i and iv by chance} = \frac{N_i N_{iv}}{N_E}$$

Equation 1b: copies/droplet of full-length transcripts with targets i and iv linked:

$$\lambda_{\text{i+iv}} = ln(N_{total}) - ln\left(N_E + N_i + N_{iv} + \frac{N_i N_{iv}}{N_E}\right)$$

Equation 1c: copies/droplet of target i without considering linkage:

$$\lambda_{\text{i}} = ln(N_{total}) - ln(N_{not\ i})$$

Equation 1d: copies/droplet of target iv without considering linkage:

$$\lambda_{\text{iv}} = ln(N_{total}) - ln(N_{not\ iv})$$

Equation 1e: percentage of linked targets obtained over the average of both targets:

$$\%\ \text{intact} = \frac{\lambda_{i+iv}}{0.5(\lambda_i + \lambda_{iv})} \times 100\%$$

Standard curves

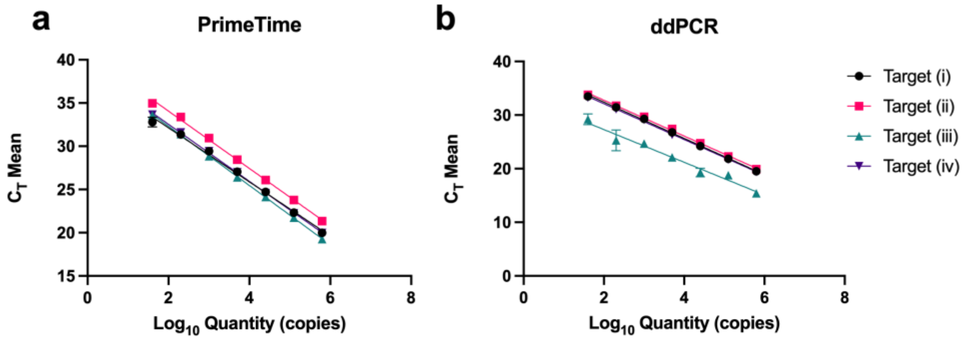

c

Target	PrimeTime Master mix	ddPCR Supermix
i	0.9931	0.9988
ii	0.9959	0.9947
ii	0.9989	0.9455
iv	0.9990	0.9974

Figure S1. Standard curves of 1:5 serial dilution of linearized saRNA fLuc plasmid using the PrimeTime (A) and ddPCR (B) master mixes. (C) Coefficient of determination (R^2) obtained for each targets' standard curve in the respective master mixes.

Capillary gel electrophoresis of saRNA-fLuc and mRNA-fLuc

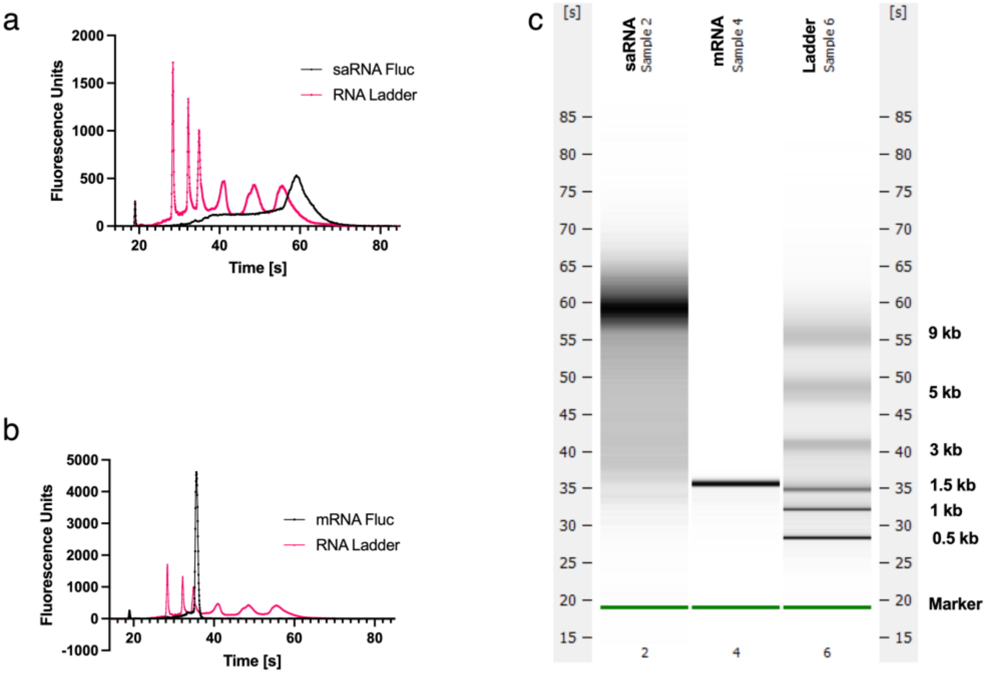

Figure S2. Sizing of shorter mRNA and long saRNA. (A) Electrogram of saRNA and RNA ladder showing migration time of the saRNA peak relative to the 9 kb peak of the ladder. (B) Electrogram of mRNA and RNA ladder. (C) Gel images of saRNA, mRNA and RNA ladder depicting sharpness of each band at their respective positions.

R code for Bioanalyzer data

```
#### Libraries ####
require(pracma)
#####

#### Constants ####
# Time points for RNA peak (numerator)
# Generally 54-64sec for saRNA
start_time = 54;
end_time = 64;

# Time points for total area (denominator)
start_time_total = 20;
end_time_total = 84.95;
#####

# Import data
# Must use original csv file with unedited row numbers
if (interactive() && .Platform$OS.type == "windows")
  data = head(read.csv(choose.files(default = "", caption = "Select Bioanalyz
er csv file",
               multi = FALSE), skip=17), -2);

# Area calculation
# Use time points where bioanalyzer peaks
# output as decimal percentage
purity = trapz(as.numeric(data$Time)[data$Time >= start_time & data$Time <= e
nd_time],
               as.numeric(data$Value)[data$Time >= start_time & data$Time <=
end_time])/
          trapz(as.numeric(data$Time)[data$Time >= start_time_total & data$Ti
me <= end_time_total],
                as.numeric(data$Value)[data$Time >= start_time_total & data$T
ime <= end_time_total])
```

Robustness of RT-ddPCR

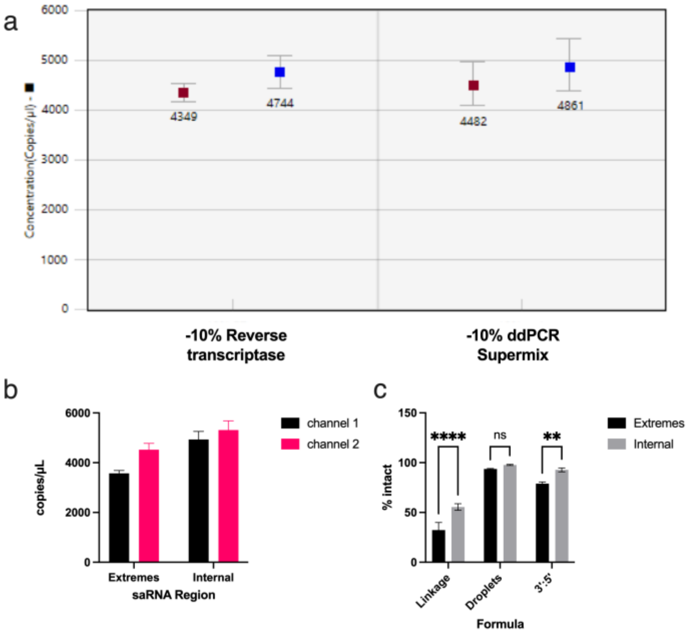

Figure S3. Assessing the resistance of RT-ddPCR to perturbations. (A) concentration of saRNA determined in assays with a 10% decrease in the final amount of reverse transcriptase and supermix. Results show similar performance to the optimized reactions. (B) Concentration of saRNA using primers/probes for the extreme and internal regions of the transcript i.e., target i + iv and targets ii and iii. (C) Percentage of intact saRNA transcripts based on concentrations obtained when probing for extreme and internal regions.

Linearity and sensitivity of ddPCR

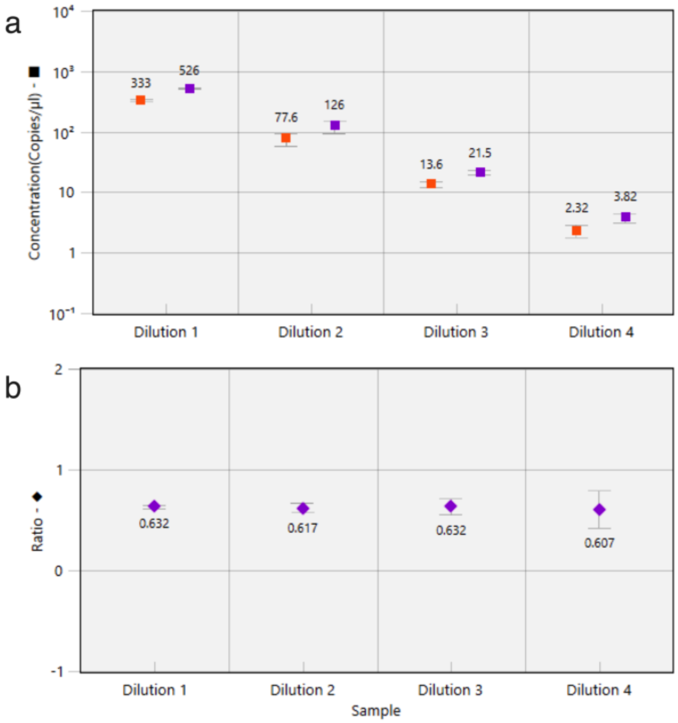

Figure S4. Linear regression and sensitivity of the two-step RT-ddPCR assay using the PS_RH strategy. (A) The log10-transformed copies/μL of cDNA generated from saRNA using the ProtoScript kit with random hexamers. The estimated Pearson correlation coefficient of the regression curve of targets (i) and (iv) are 0.999 (R^2 = 0.998, P = 0.001) and 0.9989 (R^2 = 0.9978, P = 0.0011), respectively. (B) The 3':5' ratio of each serial dilution indicating the reproducibility and reliability of independent ddPCR assays.

Amplitude multiplexing of heat degraded samples.

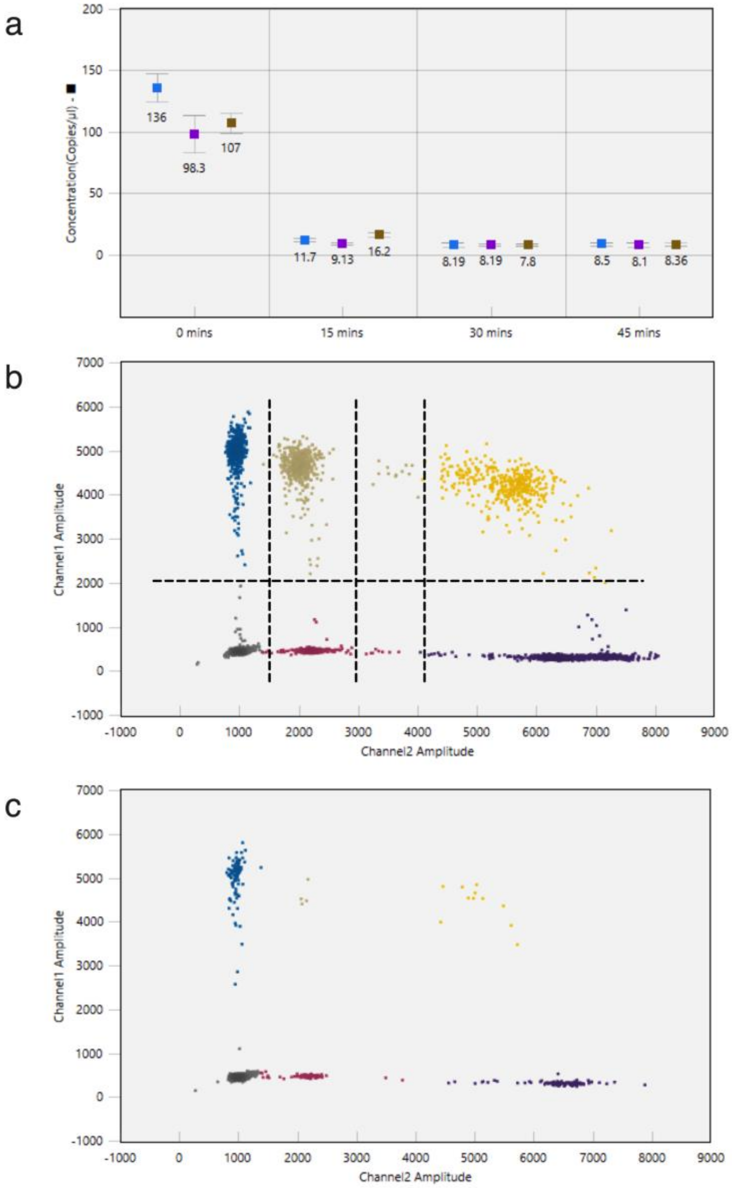

Figure S5. Two-step triplex ddPCR assay on heat degraded samples. (A) Absolute quantification of transcripts present using an amplitude multiplex assay of target I, ii and iv. (B, C) A 2D amplitude plot for the triple-plex assay of intact (B) and 45-min heat degraded saRNA(C). The droplet clusters are classified as follows: bottom left for $i^-ii^-iv^-$ droplets, top left for $i^+ii^-iv^-$ droplets, top centre-right for $i^+ii^+iv^-$ droplets, bottom centre-right for $i^-ii^+iv^-$ droplets, top

centre-left for $i^{+}ii^{-}iv^{+}$ droplets, bottom centre-right for $i^{-}ii^{-}iv^{+}$ droplets, bottom right for $i^{-}ii^{+}iv^{+}$ droplets, and top right for $i^{+}ii^{+}iv^{+}$ droplets.

Absolute quantification of glyceraldehyde-3-phosphate dehydrogenase (GAPDH)

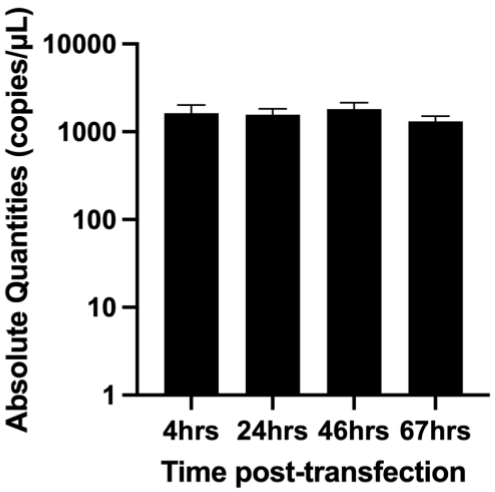

Figure S6. Absolute quantification of GAPDH mRNA levels in Lenti-X 293T cells. Concentration of GAPDH at different time points post saRNA transfection. GAPDH copy numbers were used to normalize the replication levels of saRNA-fLuc.

Original, unprocessed image of Figure 4b

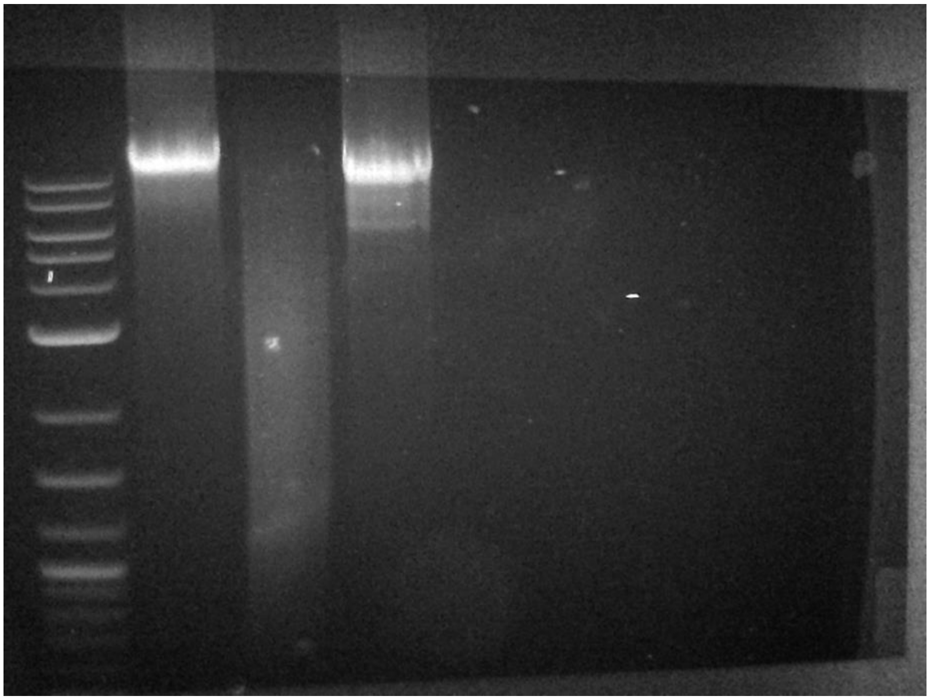

Figure S7. Original, unprocessed image of cDNA generated using different reverse transcription strategies.

Original, unprocessed image of Figure 5a

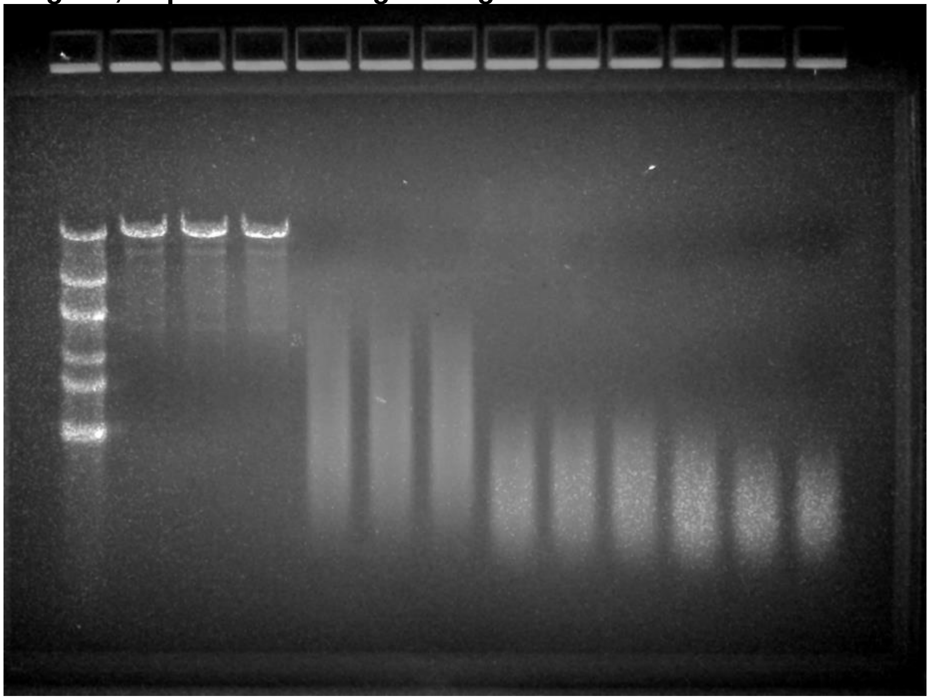

Figure S8. Original, unprocessed image of heat-degraded saRNA.
